# Use and perception of telemedicine in people with type 1 diabetes during the COVID‐19 pandemic—Results of a global survey

**DOI:** 10.1002/edm2.180

**Published:** 2020-08-29

**Authors:** Sam N. Scott, Federico Y. Fontana, Thomas Züger, Markus Laimer, Christoph Stettler

**Affiliations:** ^1^ Department of Diabetes, Endocrinology, Nutritional Medicine and Metabolism Bern University Hospital University of Bern Bern Switzerland; ^2^ Team Novo Nordisk Professional Cycling Team Atlanta GA USA; ^3^ Department of Management, Technology, and Economics ETH Zurich Zurich Switzerland

**Keywords:** COVID‐19, telemedicine, type 1 diabetes

## Abstract

**Introduction:**

The COVID‐19 pandemic has forced rapid reconsideration as to the way in which health care is delivered. One potential means to provide care while avoiding unnecessary person‐to‐person contact is to offer remote services (telemedicine). This study aimed to (1) gather real‐time information on the use and perception of telemedicine in people living with type 1 diabetes and (2) assess the challenges, such as restricted access to health care and/or medical supplies.

**Methods:**

An anonymous questionnaire was widely distributed between 24 March and 5 May 2020 using an open‐access web‐based platform. Data were analysed descriptively, and results were stratified according to age, sex and HbA_1c_.

**Results:**

There were 7477 survey responses from individuals in 89 countries. Globally, 30% reported that the pandemic had affected their healthcare access due to cancelled physical appointments with their healthcare providers. Thirty‐two per cent reported no fundamental change in their medical follow‐up during this period, with 9% stating that no personal contact was established with their doctors over the duration of the study. Twenty‐eight per cent received remote care through telephone (72%) or video‐calls (28%). Of these, 86% found remote appointments useful and 75% plan to have remote appointments in the future. Glucose control, indicated by HbA_1c_, was positively associated with positive perception of telemedicine. In males, 45% of respondents with an HbA_1c_ > 9% rated telemedicine not useful compared to those with lower HbA_1c,_ while 20% of females with an HbA_1c_ > 9% rated it not useful (χ^2^ = 14.2, *P *= .0016).

**Conclusion:**

Remote appointments have largely been perceived as positive in people with type 1 diabetes with the majority (75%) stating that they would consider remote appointments beyond the pandemic. Age and level of education do not appear to influence perception of telemedicine, whereas poor glucose control, particularly in males, seems to negatively affect perception.

## Graphical abstract

This study aimed to gather information on the use and perception of telemedicine in people living with type 1 diabetes during the COVID‐19 pandemic. An anonymous questionnaire was widely distributed. Data were analysed descriptively and the mean population responses were summarised and stratified based on country, age, sex, health status and HbA_1c_. Age and level of education do not appear to influence perception of telemedicine, whereas poor glucose control, particularly in males, seems to negatively affect perception.

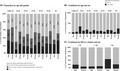

## 1 INTRODUCTION

The coronavirus disease 2019 (COVID‐19) pandemic has forced rapid reconsideration as to the way in which health care is delivered during these challenging times.^1^, ^2^ One potential means to provide care while avoiding unnecessary person‐to‐person contact is for healthcare providers to offer remote services (telemedicine) whereby appointments are conducted using digital solutions (eg phone or video call).^2^, ^3^, ^4^ Type 1 diabetes may be particularly well suited to telemedicine, as consultations are mostly based around a review of glucose data and conversations about therapy.^5^ The increasing use of continuous glucose monitoring (CGM), insulin pumps and smart insulin pens, alongside cloud/screen‐based data sharing and greater access to webcams, can make this particularly useful, as both the healthcare provider and patient can simultaneously view the data without being together physically. Virtual visits also avoid the costs, time and inconveniences of travel, which can be especially useful for people who live far from their healthcare providers or who have mobility issues that make in‐person appointments difficult.

Before the pandemic, it was thought that telemedicine approaches would only become established if long‐term studies were able to demonstrate significant time and cost savings.^6^ Commonly reported issues included increased clinician workload, data safety concerns and technical issues with equipment.^6^, ^7^ However, the pandemic has led to fast and widespread adoption of telemedicine, with the need for patient care overruling previous reservations. This study aimed to gather real‐time information on the use and perception of telemedicine in people living with type 1 diabetes during the COVID‐19 pandemic. Second, we assessed the challenges, such as restricted access to health care and/or medical supplies, and the perception of risk in this population. These data will provide improved understanding of behaviours and perception relating to remote health care during the pandemic.

## 2 METHODS

An anonymous questionnaire was widely distributed via social media (Twitter, Facebook and Instagram) on a weekly basis between 24 March and 5 May 2020 using an open‐access web‐based platform (SurveyMonkey.com; Figure S1). The survey covered questions relating to the use and perception of telemedicine, diabetes treatment and control, and medical supply during the pandemic. The questionnaire was available in English, Spanish, German, French and Italian. Data were analysed descriptively, and results were stratified according to age (<18, 18‐24, 25‐34, 35‐44, 45‐54, 55‐64, >65 years), sex and HbA_1c_ (<7.0%, 7%‐9%, >9%). After assumption verification, categories were compared using independent t tests, one‐way and two‐way ANOVAs with post hoc multiple comparisons (Tukey's HSD) and Pearson's chi‐square (χ^2^) tests over contingency tables. Outliers were checked using z‐scores, expecting 5% of scores above 1.96, 1% above 2.58 and none above 3.29. In the case of outliers’ detection, raw data were re‐checked to exclude any source of error in the measurement before data points were removed. In case of missing data, pairwise exclusion was applied. All statistical analyses were performed using RStudio (Version 1.1.447, 2018, Boston, USA), and α was set at 0.05; statistical significance was accepted when *P *< α.

## 3 RESULTS

There were 7477 survey responses from individuals in 89 countries (37% from Europe, North America 37%, South America 4%, Oceania 5% and 17% from Africa and Asia) over the 7 weeks of data collection. The majority of responses were collected from the USA (33%) and UK (15%). Sixty‐eight per cent of respondents were women, and 32% were men. Fifty‐three per cent of respondents were in the 25‐44 years of age category, and the average HbA_1c_ of the entire cohort was 7.1 ± 1.2%. The average time since diagnosis was 17 ± 12 years. Fifty‐six per cent were on insulin pump therapy, 43% used multiple daily injections, and 1% used both.

The majority (85%) perceived themselves to be in a good‐to‐excellent pre‐existing health condition. However, 45% reported to have additional medical complications besides type 1 diabetes with thyroid disease (27%) and asthma (10%) being the most reported. Forty‐two per cent reported to have concomitant diabetes complications such as mild to severe retinopathy (18%), diabetic neuropathy (6%) and cardiovascular disease (16%). Moreover, 11% experienced respiratory complications requiring medications over the last 6 months with asthma (37%), bronchitis (9%) and pneumonia (7%) being the most reported.

**Figure 1:**
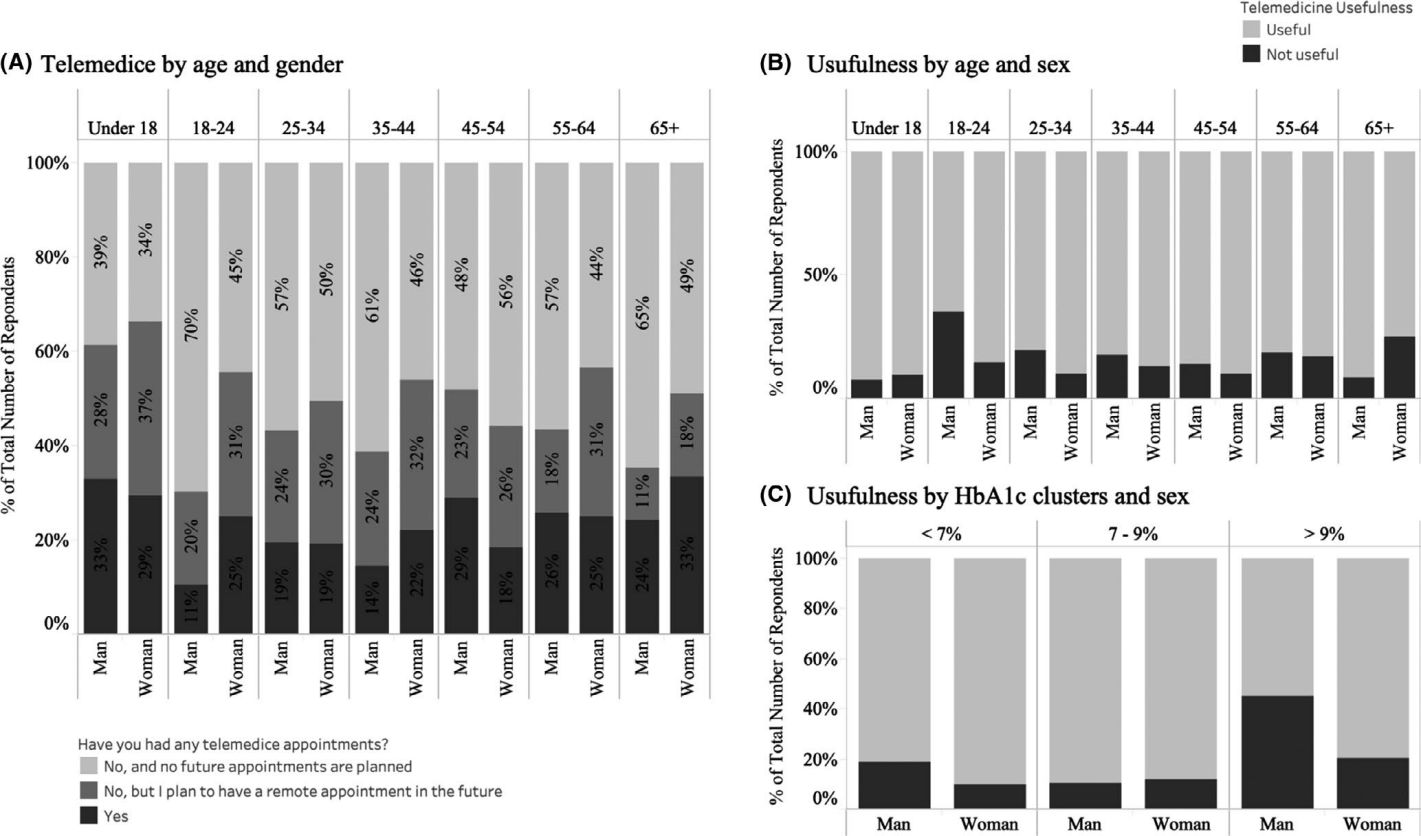
Use and perception of telemedicine by survey respondents. A, Proportion of responders stating that they have used or intend to use telemedicine. Perception of telemedicine according to age and sex (B) and according to HbA_1c_ and sex (C)

Globally, 30% reported that the pandemic had affected their healthcare access due to cancelled appointments with their healthcare providers. Thirty‐two per cent reported no fundamental change in their medical follow‐up during this period, with 9% stating that no personal contact was established with their doctors over the duration of the study. Twenty‐eight per cent received remote care through telephone (72%) or video‐calls (28%). Of these, 86% found remote appointments useful and 75% plan to have remote appointments in the future (Figure 1). Glucose control indicated by HbA_1c_ was associated with perception of telemedicine (Figure 1C). In males, 45% of respondents with an HbA_1c_ > 9% rated telemedicine useless compared to those with lower HbA_1c,_ while 20% of females with an HbA_1c_ > 9% rated it not useful (χ^2^ = 14.2, *P* = .0016). Overall, 79% of respondents reported no issues in accessing diabetes supplies and medications. Insulin (8%), continuous glucose monitors (6%) and fast‐acting carbohydrates (6%) were the diabetes‐related supplies most difficult to access due to the COVID‐19 pandemic so far.

## 4 DISCUSSION

The data from this worldwide survey demonstrate that a large percentage of respondents with type 1 diabetes either have had or plan to have remote diabetes consultations as a result of the COVID‐19 pandemic. These remote appointments have largely been perceived as positive with the majority (75%) stating that they would consider remote appointments beyond the pandemic. There was no difference in perception between age groups or level of educational background, suggesting that telemedicine may be useful for a large proportion of the population. However, respondents, particularly males, with poor glycaemic control (HbA_1c_ > 9%) were more likely to rate telemedicine as not useful.

Due to the rapidly changing situation at the start of the pandemic, there was tremendous uncertainty, with almost no information on how people with type 1 diabetes would be affected. At the time, it was unknown whether people would experience difficulties gaining access to supplies such as insulin or what the impact of missing appointments with their endocrinologist would mean. People with type 1 diabetes are reliant on access to medical supplies such as insulin and blood glucose monitoring equipment. Limited access to healthcare providers and medical supplies can be of particular concern during such stressful situations and in patients with poor glycaemic control, as this is powerfully associated with increased risk of infection and other negative outcomes.^8^ In those that responded to this survey, 30% reported that the pandemic had affected their healthcare access due to cancelled appointments with their healthcare providers.

While discussions about remote telemedicine are rapidly increasing due to the pandemic, a number of studies have previously investigated the potential benefits of telemedicine in diabetes care.^6^, ^9^, ^10^ In a systematic review of 29 studies in the field of paediatric diabetes care,^7^ it was concluded that telemedicine has the potential to facilitate patient monitoring and can improve short‐term glycaemic control in some contexts. It has also been suggested that virtual diabetes care may prove effective in aiding behavioural change, as well as providing social and psychological support.^11^ However, commonly reported issues included increased clinician workload, data safety concerns and technical issues with equipment. These findings were in line with those of Frielitz et al,^12^ who interviewed 9 diabetes telemedicine experts to investigate the healthcare professional's perceptions of telemedicine. They also found that the healthcare professionals see the benefits of telehealth and video counselling, with the caveat that technical issues and staff workload can be potential issues. On the other hand, when there is no possibility to go for a physical appointment, as during the pandemic, reservations against telemedicine may be reduced, as suggested by the present analysis.

A recently published perspective article highlighted how paediatric patients with type 1 diabetes have historically led the way in the adoption of diabetes technology.^13^ In their article, Danne and Limbert^13^ identify how young patients have been particularly receptive to new technologies such as insulin pumps and glucose sensors, suggesting the trend would continue for telemedicine. Interestingly, according to our data, perception of telemedicine was not different between age groups or level of educational background, suggesting that telemedicine may be useful for a large proportion of the population and not just in paediatric patients. Here we also collected data from a large number of respondents in the >65 years of age bracket, with most viewing telemedicine positively. This is encouraging as it suggests telemedicine is useful for a wide variety of individuals and not just for younger patients.

The only factor that did influence perception of telemedicine in our data set was HbA_1c_, whereby poor glucose control (>9%), particularly in males, was associated with negative perception of telemedicine. Based on the data we have, it is not possible to infer cause and effect. This lower perception of telemedicine could be because these individuals are less likely to be motivated to engage in this type of care or that they are frustrated that they do not experience greater improvements with telemedicine. Beyond the pandemic, it may be useful to offer telemedicine to patients based on how useful it is for a given individual. The difference in perceptions between males and females shown here cannot be explained but at least strongly hint towards a high acceptance also of the female population. In this context, it is also interesting to note that in contrast to many scientific reports and studies, 68% of respondents were female vs. 32% male in this study, again corroborating the appreciation of female patients for this important topic.

The strength of this study has been our ability to quickly obtain a large number of responses from individuals around the world. Fast knowledge gathering and dissemination is crucial during a highly dynamic situation such as the current COVID‐19 pandemic, to facilitate access to healthcare equipment and resources to those that need them most. However, we acknowledge a number of limitations. Our data are limited to those that completed the survey and thus those with access to the Internet. It may, therefore, not be representative of everyone with type 1 diabetes, and there are likely to be social biases, although we tried to control for this by collecting information on demographics. Second, it is difficult to make direct comparisons between countries with our data set because there are several countries with a low number of responses (eg in South America and Oceania compared to North America and parts of Europe) that are not representative of the population response and reaction. For this reason, data were analysed with caution and interpreted as a unique sample, representative of the population of interest as a whole.

In conclusion, the COVID‐19 pandemic poses unique challenges to diabetes care. The results from this real‐time worldwide survey demonstrate that a large number of people living with type 1 diabetes have rapidly adopted telemedicine or plan to in the near future and that this has generally been perceived positively. Interestingly, age and level of education do not appear to influence peoples’ perceptions of telemedicine so far, whereas poor glucose control seems to negatively affect the perception on usefulness of telemedicine, particularly in males. Beyond the pandemic, telemedicine may offer an alternative means to improve efficiency and cost effectiveness of care for people with diabetes.

## CONFLICT OF INTEREST

The authors have no conflicts of interest to disclose.

## AUTHOR CONTRIBUTIONS

All authors contributed to the design of the study, data collection, analysis and interpretation of the results. SNS, FYF, TZ and CS prepared the first draft of the manuscript and all authors reviewed and approved the manuscript. CS is the guarantor of this work and, as such, had full access to all the data in the study and takes responsibility for the integrity of the data and the accuracy of the data analysis.

## ETHICAL APPROVAL

The study was performed in accordance with the Declaration of Helsinki and the regulations of the local ethical committee.

## Supporting information

Supplementary Material

## Data Availability

The data that support the findings of this study are available from the corresponding author upon reasonable request.
