# Supplementary material for: Use and perception of telemedicine in people with type 1 diabetes during the COVID‐19 pandemic—Results of a global survey
Source: Endocrinol Diabetes Metab. 2020 Aug 29;4(1):e00180. doi: 10.1002/edm2.180 (PMC7831200; doi:10.1002/edm2.180)
Supplement: Supplementary file 1 — Supplementary Material [file EDM2-4-e00180-s001.pdf]

Week 6

We currently have limited information on whether there are additional risks for people living with type 1 diabetes during the COVID-19 pandemic.
Your responses to this questionnaire will help researchers and medical professionals from around the world to adapt and develop strategies to better respond and provide medical treatment.

The questionnaire will be updated weekly. Please consider completing the questionnaire again every week so that we can track the progress throughout the COVID-19 pandemic.
We value your privacy. At the end of the questionnaire, you will be asked to provide your email address. This is not mandatory and we will never share your email address with anyone. Additionally, your answers will always remain anonymous. We will only use your email address (if provided) to keep you updated every time a new weekly survey becomes available.
Survey results will feed an interactive dashboard that will be public and updated weekly. Your data will be completely anonymised before public sharing.
For any request please contact: T1DCOVID19study@gmail.com

* Do you agree to the above terms? By clicking Yes, you consent that you are willing to answer the questions in this survey

- ◯ Yes
- ◯ No

Here you can access SurveyMonkey's privacy policy to see how they handle respondent data on our behalf

* How old are you?

- Under 18
- 18-24
- 25-34
- 35-44
- 45-54
- 55-64
- 65+

* What is your gender?

- Man
- Woman

* What is the highest level of school you have completed or the highest degree you have received?

- Less than high school degree
- High school degree or equivalent
- Some college but no degree
- Bachelor degree
- Graduate degree
- Postgraduate degree

* In what country do you live?

* How long ago were you diagnosed with type 1 diabetes?

* What was your most recent HbA1c (%)?

* What's your current therapy regimen?

- [ ] Multiple daily injections
- [ ] Insulin pump therapy
- [ ] Both

* How would you rate your general health?

* Do you have any additional medical conditions besides type 1 diabetes?

- ○ Yes
- ○ No

If yes, please specify (optional)

[ ]

* Did you have any respiratory conditions requiring medication over the last 6 months?

- ○ Yes
- ○ No
- ○ Don't remember

If yes, please specify condition (if known)

[ ]

* Do you have any of the following complications?

- ☐ No complications to report
- ☐ Peripheral vascular disease (e.g. impairment of circulation in limbs/feet)
- ☐ Cardiac disease (e.g. coronary heart disease, heart attack, heart failure or similar)
- ☐ Cerebrovascular disease (stroke or similar)
- ☐ High blood pressure
- ☐ Mild retinopathy (eye disease)
- ☐ Severe retinopathy
- ☐ Diabetic neuropathy (impairment of nerve function)
- ☐ Diabetic nephropathy (albuminuria / renal disease)
- ☐ Don't know
- ☐ Other (please specify)

[ ]

* Has the current COVID-19 pandemic affected your access to healthcare?

- ○ Yes, my regular diabetes-related appointments have all been cancelled
- ○ No, everything is running as usual
- ○ I decided to cancel my regular appointments
- ○ I have spoken to my healthcare provider over the phone or via other remote means
- ○ I haven't spoken to my healthcare provider yet

Other (please give details)

[ ]

* Have you had any appointments related to your diabetes with your healthcare provider/endocrinologist remotely since the beginning of the COVID-19 pandemic?

- No, and no future appointments are planned
- No, but I plan to have a remote appointment in the future
- Yes

If you have had a remote appointment with your healthcare provider/endocrinologist since the start of the pandemic, how useful did you find this?

No responses needed if you didn't have any remote appointments.

- Extremely useful
- Very useful
- Somewhat useful
- Not so useful
- Not at all useful

If you have had a remote appointment with your healthcare provider/endocrinologist, how was this done?

No responses needed if you didn't have any remote appointments.

- Telephone
- Video call e.g. skype/zoom
- Other (please specify)

* How likely are you to consider remote appointments instead of appointments in person after the pandemic?

- Very likely
- Likely
- Neither likely nor unlikely
- Unlikely
- Very unlikely

* Has the COVID-19 pandemic affected your level of access to the following?

- Insulin
- Needles
- Continuous glucose sensors/flash glucose sensors
- Blood glucose metres
- Testing strips
- Glucagon
- Fast-acting carbohydrates to treat hypoglycaemia
- Other diabetes medication
- No issues to report
- Other (please specify)

* Since the start of the pandemic have you experienced a change in your general glucose control indicated by any of the following:

- [ ] Frequency of **hypoglycemia** (glucose values < 70mg/dl / < 3.9 mmol/L) decreased
- [ ] Frequency of **hypoglycemia** (glucose values < 70mg/dl / < 3.9 mmol/L) increased
- [ ] Frequency of **hyperglycemia** (glucose values > 180mg/dl / > 10mmol/L) decreased
- [ ] Frequency of **hyperglycemia** (glucose values > 180mg/dl / > 10mmol/L) increased
- [ ] HbA1c increased
- [ ] HbA1c decreased
- [ ] No changes observed
- [ ] I don’t know

* If you are using a continuous glucose monitor or flash glucose monitor, have you noticed any of the following since the start of the pandemic?

- [ ] The time in target range (70-180 mg/dl / 3.9 – 10 mmol/L) of sensor-glucose decreased
- [ ] The time in target range (70-180 mg/dl / 3.9 – 10 mmol/L) of sensor-glucose increased
- [ ] Time below target range (< 70mg/dl / < 3.9 mmol/L) of sensor-glucose decreased
- [ ] Time below target range (< 70mg/dl / < 3.9 mmol/L) of sensor-glucose increased
- [ ] Time above target range (> 180mg/dl / >10mmol/L) of sensor-glucose decreased
- [ ] Time above target range (> 180mg/dl / >10mmol/L) of sensor-glucose increased
- [ ] No changes observed
- [ ] I don’t know
- [ ] I am not using a continuous glucose monitor or flash glucose monitor

* Have you been tested for COVID-19?

- ○ Yes, and it was positive
- ○ Yes, and it was negative
- ○ No

* If you tested positive for COVID-19 were you hospitalised?

- No
- Yes
- Yes, and have since been discharged from hospital

* If you tested positive for COVID-19, what were your symptoms?

- I haven't had symptoms
- Mild cough
- Severe cough
- Sneezing
- Fever
- Tiredness
- Mild breathing difficulty
- Severe breathing difficulty
- High temperature
- Gastrointestinal symptoms

Other (please specify)

* If you have had symptoms, how long did they last?

- I have not had symptoms
- 1-2 days
- 3-4 days
- 4-5 days
- 6-7 days
- 7-10 days
- More than 10 days
- Don't remember

* If you have or have had COVID-19, how did it affect your glucose control?

- Not at all
- Slightly
- Somewhat
- Moderately
- Substantially/Significantly
- Please provide details (optional)

* Do you feel as though you are at higher risk during the COVID-19 pandemic than the average person because you have type 1 diabetes?

- ◯ Yes
- ◯ No
- ◯ Don't know

Other (please specify)

* How strongly do you agree with the following statement: 'My healthcare system is supporting me in my diabetes management during the COVID-19 pandemic'

- ◯ Don't know
- ◯ Strongly agree
- ◯ Agree
- ◯ Neutral
- ◯ Disagree
- ◯ Strongly disagree

Thank you for taking the time to complete this questionnaire. Your responses are extremely valuable to us.
They will allow researchers and medical professionals to adapt during the COVID-19 pandemic.
Please consider completing the questionnaire again next week so that we can track the progress throughout the COVID-19 pandemic.

Send me a reminder to complete the questionnaire again next week.

Email Address ____________________

* As a resident of countries affected by GDPR, we are required to request your consent and acknowledgement that you authorise us to store your email address.
Do you consent with your personal data being processed as described in the intro page?

- [ ] Yes
- [ ] No
